# Nomogram based on the novel index LANR, composed of preoperative lymphocytes, albumin, and neutrophils, for predicting prognosis in patients with gastric cancer: a retrospective study

**DOI:** 10.3389/fonc.2025.1634948

**Published:** 2025-09-29

**Authors:** Ruoyun Li, Qinghua Liu, Haohao Wang, Qingjie Chen, Chaofan Pan, Wenbin Luo, Changjiang Luo

**Affiliations:** Department of General Surgery, Lanzhou University Second Hospital, Lanzhou, China

**Keywords:** gastric cancer, LANR, prognosis, overall survival, nomogram

## Abstract

**Purpose:**

This study aims to explore the relationship between the novel index LANR, which is composed of preoperative lymphocytes, neutrophils, and albumin, and prognosis in patients with gastric cancer (GC), and to develop and visualize a new nomogram for predicting overall survival (OS) in GC patients.

**Methods:**

A total of 497 patients (346 in the training cohort and 151 in the validation cohort) with GC who underwent radical resection were retrospectively analyzed. The LANR was calculated as the lymphocyte×albumin/neutrophil. Collinearity diagnostic analysis was performed to assess the correlations between variables. Univariate and multivariate Cox regression analyses were used to identify independent prognostic factors for OS, which were then used to construct a nomogram model. The efficacy of the nomogram was subsequently evaluated in the validation cohort.

**Results:**

Multivariate Cox regression analysis showed that tumor size (Hazard ratio [HR]=1.653, P = 0.001), T stage (HR = 3.236, P<0.001), N stage (HR = 2.059, P<0.001), chemotherapy (HR = 1.508, P = 0.005), and LANR (HR = 0.586, P<0.001) were independent significant risk factors for OS in patients with GC. The independent prognostic performance of LANR is superior compared to NLR, PNI and PLR. In the training cohort, the area under the curve (AUC) of the nomograms for predicting 3-, 5- and 7-year OS were 0.768(95% CI = 0.718–0.819), 0.832(95% CI = 0.790–0.875) and 0.893(95% CI = 0.830–0.956), respectively. The AUC of the nomogram for predicting 3-, 5- and 7-year OS were 0.795(95% CI = 0.719–0.871), 0.823(95% CI = 0.756–0.890) and 0.833(95% CI = 0.735–0.931), respectively, in the validation cohort. Both in the training and validation cohorts, the calibration curves showed good consistency between the actual survival rates and the predicted values from the nomogram. The Decision curve analysis also indicated that the model has clinical utility.

**Conclusion:**

LANR is an independent prognostic factor for GC. The newly developed nomogram demonstrates high accuracy and potential clinical utility in predicting the OS in GC patients.

## Introduction

Gastric cancer (GC) is among the most prevalent malignant tumors of the digestive system. Global cancer statistics indicate that in 2021, there were 968,350 new cases and 659,853 deaths due to gastric cancer worldwide (1). The insidious onset, high invasiveness, and malignant nature of GC lead most patients to be diagnosed at intermediate or advanced stages, resulting in a poor prognosis (2, 3). The TNM staging system is widely used for clinical prognosis evaluation (4); however, due to the heterogeneity of GC, clinical outcomes can vary significantly, even among patients at the same TNM stage receiving similar treatments (5). Therefore, it is crucial to identify more precise biomarkers for predicting the prognosis of GC.

With advancements in cancer prognosis research, evidence increasingly suggests that inflammatory responses and nutritional status are crucial (6, 7). Inflammation can promote tumor growth, invasion, and metastasis (8), whereas nutritional status reflects overall health and immune function (9). Inflammatory markers, such as the neutrophil-to-lymphocyte ratio, platelet-to-lymphocyte ratio, and pan-immune-inflammation value, are confirmed reliable prognostic biomarkers for GC patients (10, 11). Additionally, markers reflecting nutritional status, such as C-reactive protein, albumin (ALB) levels, and the prognostic nutritional index, also predict GC prognosis (12, 13). However, individual inflammatory and nutritional markers cannot comprehensively reflect the body’s status, limiting their prognostic value. LANR is a novel biomarker composed of lymphocytes, ALB, and neutrophils. By integrating inflammation, immunity, and nutritional status, it offers a more comprehensive tool for prognostic assessment. Existing studies show that LANR holds independent prognostic value in evaluating pancreatic cancer (14), nasopharyngeal carcinoma (15), and colorectal cancer (16). However, the prognostic significance of LANR in GC patients remains unexplored.

This study aims to use preoperative LANR, reflecting inflammation, immunity, and nutritional status, to explore its relationship with prognosis in GC patients. Additionally, it seeks to develop a new nomogram predictive model for effectively predicting overall survival (OS) in GC patients.

## Materials and methods

### Study population

A retrospective analysis was conducted on GC patients who underwent radical resection at Lanzhou University Second Hospital from January 2017 to June 2019. Inclusion criteria were: (1) pathologically diagnosed with GC; (2) complete clinical, pathological, laboratory, and follow-up data; (3) no distant metastasis. Exclusion criteria included: (1) preoperative neoadjuvant therapy; (2) a history of hematological diseases, chronic inflammatory conditions, or malignant tumors. All enrolled patients were randomly assigned in a 7:3 ratio to either the training cohort (n = 346) or the validation cohort (n = 151). This study followed the principles of the Declaration of Helsinki and was approved by the Ethics Committee of Lanzhou University Second Hospital (Project Number:2025 A-028). Given that all data were anonymized and patient privacy was protected, the Ethics Committee waived the requirement for informed consent.

### Data collection

Data for all patients were obtained from the hospital’s medical records system, which provided demographic, laboratory, and clinical pathological information, including gender, age, neutrophil count, lymphocyte count, serum albumin (ALB), Platelet count, tumor location, grade, tumor size, T stage, N stage, and TNM stage (based on the 8th edition of the American Joint Committee on Cancer (AJCC) TNM staging system). Based on these data, composite hematological indices LANR, NLR, PNI and PLR were calculated.

### Follow-up

Through telephone interviews or review of inpatient and outpatient medical records, with the last follow-up date being July 1, 2024. OS was defined as the time from the date of surgery to the date of death from any cause or to July 1, 2024.

### Statistical analysis

Data analysis was performed using SPSS (version 26.0) and R software (version 4.3.2). The optimal cut-off values for LANR and risk stratification were determined using X-tile (version 3.6.1), which maximizes log-rank separation in censored survival data (17). The chi-square test was applied to analyze the association between LANR and clinical pathological features. Survival curves were generated using the Kaplan-Meier method, and the log-rank test was applied. Multicollinearity was assessed using the variance inflation factor (VIF). VIF values < 5 indicated no substantial multicollinearity. Univariate and multivariate analyses were conducted using the Cox proportional hazards model to identify independent prognostic factors, with two-sided P-values < 0.05 considered statistically significant. Receiver operating characteristic (ROC) analysis was used to compare the prognostic performance of LANR with that of other composite indices. Based on the results of the multivariate Cox regression analysis, a nomogram model for predicting OS was developed. Calibration curves were used to assess the calibration of the clinical prediction model. Decision curve analysis (DCA) was applied to evaluate the clinical utility of the nomogram. The predictive performance of the nomogram model was assessed using the concordance index (C-index), ROC curves, and the area under the curve (AUC).

## Results

### Patient characteristics

A total of 497 GC patients were included in this study. Among them, 77.9% were male, 22.1% were female, 51.7% were younger than 60 years, and 48.3% were 60 years or older. The median follow-up time for the entire cohort was 51 months, with 3-year and 5-year OS rates of 63.6% and 43.9%, respectively. Detailed Demographic and clinical characteristics of the training cohort (n = 346) and the validation cohort (n = 151) are presented in **Table 1**. No statistically significant differences were detected in measured baseline characteristics between the two cohorts (all P > 0.05).

**Table 1:** Demographic and clinical characteristics of patients with GC.

Variable	Total	Training cohort	Validation cohort	P
	N=497(%)	N=346(%)	N=151(%)	
Age				0.547
<60	257(51.7)	182(52.6)	75(49.7)	
≥60	240(48.3)	164(47.4)	76(50.3)	
Gender				0.921
Male	387(77.9)	269(77.7)	118(78.1)	
Female	110(22.1)	77(22.3)	33(21.9)	
Tumor location				0.786
Cardia	128(25.8)	92(26.6)	36(23.8)	
Body	106(21.3)	74(21.4)	32(21.2)	
Antrum	263(52.9)	180(52.0)	83(55.0)	
Tumor size (cm)				0.369
<5	262(52.7)	187(54.0)	75(49.7)	
≥5	235(47.3)	159(46.0)	76(50.3)	
Lauren				
Intestinal	188(37.8)	126(36.4)	62(41.0)	0.614
Diffuse	189(38.0)	135(39.0)	54(35.8)	
Mix	120(24.2)	85(24.6)	35(23.2)	
Grade				0.569
Well	45(9.0)	33(9.5)	12(8.0)	
Moderate	302(60.8)	205(59.3)	97(64.2)	
Poor	150(30.2)	108(31.2)	42(27.8)	
T stage				0.862
1-2	121(24.3)	85(24.6)	36(23.8)	
3-4	376(75.7)	261(75.4)	115(76.2)	
N stage				0.613
N0	199(40.0)	136(39.3)	63(41.7)	
N1-N3	298(60.0)	210(60.7)	88(58.3)	
TNM stage				0.892
I	106(21.3)	72(20.8)	34(22.5)	
II	114(22.9)	79(22.8)	35(23.2)	
III	277(55.8)	195(56.4)	82(54.3)	
Chemotherapy				0.228
Yes	276(55.5)	186(53.8)	90(59.6)	
No	221(44.5)	160(46.2)	61(40.4)	
LANR	15.95(10.77,24.52)	16.66(10.95,25.07)	14.77 (10.22,22.61)	0.120
NLR	2.51 (1.69,3.58)	2.48(1.66,3.51)	2.81(1.74,3.73)	0.128
PNI	48.2(44.15,51.90)	47.90(44.14,51.86)	48.3(44.15,51.95)	0.902
PLR	139.57(107.85,193.63)	139.34(103.36,194.24)	139.57(111.34,190.32)	0.572

### The relationship between LANR and clinical pathological characteristics and prognosis

**Figure 1:**
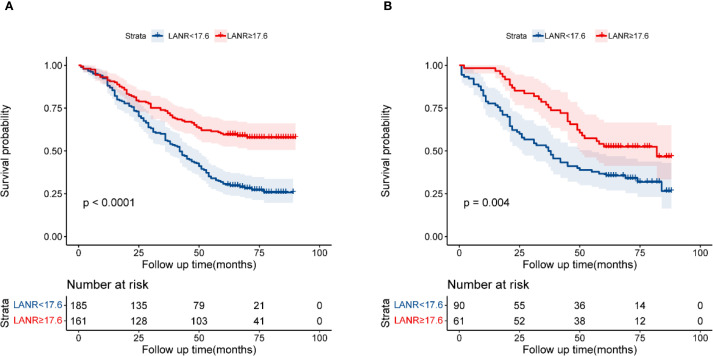
Kaplan–Meier curves of OS in patients with GC by LANR in the training set **(A)**; and the validation set **(B)**.

The optimal cutoff value for LANR was determined to be 17.6 using X-tile software. The association between LANR and the clinical pathological features of patients is presented in **Table 2**. In the training cohort, LANR was associated with age, gender, tumor size, T stage, N stage, and TNM stage. In the validation cohort, LANR was associated with T stage. In both cohorts, LANR was not associated with tumor location, Lauren, grade and chemotherapy. The Kaplan-Meier analysis and log-rank tests showed that LANR was significantly associated with OS in both the training cohort and the validation cohort (P < 0.05) (**Figure 1**).

**Table 2:** The association between LANR and the clinical pathological features of patients with GC.

Variable	Training cohort				Validation cohort			
	Low	High	χ2	P	Low	High	χ2	P
Age			8.257	0.004			0.054	0.816
<60	84(45.4)	98(60.9)			44(48.9)	31(50.8)		
≥60	101(54.6)	63(39.1)			46(51.1)	30(49.2)		
Gender			4.482	0.034			2.168	0.141
Male	152(82.2)	117(72.7)			74(82.2)	44(72.1)		
Female	33(17.8)	44(27.3)			16(17.8)	17(27.9)		
Tumor location			2.01	0.366			3.990	0.136
Cardia	55(29.7)	37(23.0)			26(28.9)	10(16.4)		
Body	38(20.6)	36(22.4)			20(22.2)	12(19.7)		
Antrum	92(49.7)	88(54.6)			44(48.9)	39(63.9)		
Tumor size (cm)			26.909	<0.001			3.577	0.059
<5	76(41.1)	111(68.9)			39(43.3)	36(59.0)		
≥5	109(58.9)	50(31.1)			51(56.7)	25(41.0)		
Lauren			0.268	0.874			2.917	0.233
Intestinal	68(36.8)	58(36.0)			32(35.6)	30(49.2)		
Diffuse	70(37.8)	65(40.4)			36(40.0)	18(29.5)		
Mix	47(25.4)	38(23.6)			22(24.4)	13(21.3)		
Grade			0.025	0.988			4.311	0.116
Well	18(9.7)	15(9.3)			4(4.4)	8(13.1)		
Moderate	109(58.9)	96(59.6)			58(64.5)	39(63.9)		
Poor	58(31.4)	50(31.1)			28(31.1)	14(23.0)		
T stage			5.596	0.018			4.511	0.034
1-2	36(19.5)	49(30.4)			16(17.8)	20(32.8)		
3-4	149(80.5)	112(69.6)			74(82.2)	41(67.2)		
N stage			5.592	0.018			0.735	0.391
N0	62(33.5)	74(46.0)			35(38.9)	28(45.9)		
N1-N3	123(66.5)	87(54.0)			55(61.1)	33(54.1)		
TNM stage			9.34	0.009			4.402	0.111
I	27(14.6)	45(28.0)			15(16.7)	19(31.2)		
II	45(24.3)	34(21.1)			22(24.4)	13(21.3)		
III	113(61.1)	82(50.9)			53(58.9)	29(47.5)		
Chemotherapy			3.337	0.068			0.047	0.828
Yes	91(49.2)	95(59.0)			53(58.9)	37(60.7)		
No	94(50.8)	66(41.0)			37(41.1)	24(39.3)		

### Identification of independent prognostic factors

In the training cohort, univariate Cox regression analysis showed that age, tumor size, grade, T stage, N stage, TNM stage, chemotherapy, and LANR were significantly associated with OS (**Table 3**). Potential multicollinearity among variables significant in the univariate Cox analyses was assessed. Results showed that the VIFs for T stage, N stage, and TNM stage all exceeded 5, suggesting multicollinearity (**Table 4**). Because TNM stage had a VIF of 16.794 and is a composite of T and N, it was not included in subsequent multivariable analyses. After excluding TNM stage, VIFs for the remaining variables were <5 (**Table 5**). The remaining variables (age, tumor size, grade, T stage, N stage, chemotherapy, and LANR) were entered into the final multivariable Cox regression model, which revealed that tumor size (Hazard ratio [HR]=1.653, P = 0.001), T stage (HR = 3.236, P<0.001), N stage (HR = 2.059, P<0.001), chemotherapy (HR = 1.508, P = 0.005), and LANR (HR = 0.586, P<0.001) were independent prognostic risk factors for OS (**Table 3**). We compared AUC values for LANR with those for NLR, PNI and PLR at 3, 5, and 7 years in both the training and validation cohorts. LANR showed higher or similar AUC values at these time points (**Supplementary Table 1** and **Supplementary Table 2**). LANR was incorporated into the final nomogram.

**Table 3:** Univariate and multivariate cox regression analysis of OS in patients with GC.

Variable	Univariate analysis	P	Multivariate analysis	P
	HR (95%CI)		HR (95%CI)	
Age				
<60				
≥60	1.570(1.188,2.073)	0.001	1.261(0.946,1.680)	0.114
Gender				
Male				
Female	1.047(0.751,1.458)	0.787		
Tumor location				
Cardia				
Body	0.947(0.643,1.394)	0.781		
Antrum	0.757(0.547,1.048)	0.094		
Tumor size (cm)				
<5				
≥5	2.934(2.200,3.914)	<0.001	1.653(1.223,2.235)	0.001
Lauren				
Intestinal				
Diffuse	1.355(0.985,1.864)	0.062		
Mix	1.068(0.738,1.545)	0.727		
Grade				
Well				
Moderate	2.267(1.222,4.205)	0.009	1.559(0.828,2.936)	0.169
Poor	2.770(1.465,5.238)	0.002	1.921(0.998,3.696)	0.051
T stage				
1-2				
3-4	5.875(3.518,9.812)	<0.001	3.236(1.875,5.586)	<0.001
N stage				
N0				
N1-N3	3.525(2.518,4.935)	<0.001	2.059(1.444,2.938)	<0.001
TNM stage				
I				
II	3.195(1.662,6.144)	<0.001		
III	8.443(4.678,15.238)	<0.001		
Chemotherapy				
Yes				
No	1.435(1.088,1.893)	0.011	1.508(1.132,2.009)	0.005
LANR				
<17.6				
≥17.6	0.459(0.342,0.616)	<0.001	0.586(0.433,0.792)	<0.001

**Table 4:** Collinearity diagnostics among the variables age, tumor size (cm), grade, T stage, N stage, TNM stage, chemotherapy, LANR.

Variable	Collinearity diagnosis	
	VIF	Tolerance
Age	1.076	0.930
Tumor size (cm)	1.312	0.762
Grade	1.070	0.935
T stage	6.569	0.152
N stage	6.563	0.152
TNM stage	16.794	0.060
Chemotherapy	1.067	0.938
LANR	1.117	0.895

**Table 5:** Collinearity diagnostics among the variables age, tumor size (cm), grade, T stage, N stage, chemotherapy, LANR.

Variable	Collinearity diagnosis	
	VIF	Tolerance
Age	1.072	0.933
Tumor size (cm)	1.289	0.776
Grade	1.068	0.937
T stage	1.401	0.714
N stage	1.324	0.755
Chemotherapy	1.065	0.939
LANR	1.117	0.896

### Construction and validation of the nomogram

**Figure 2:**
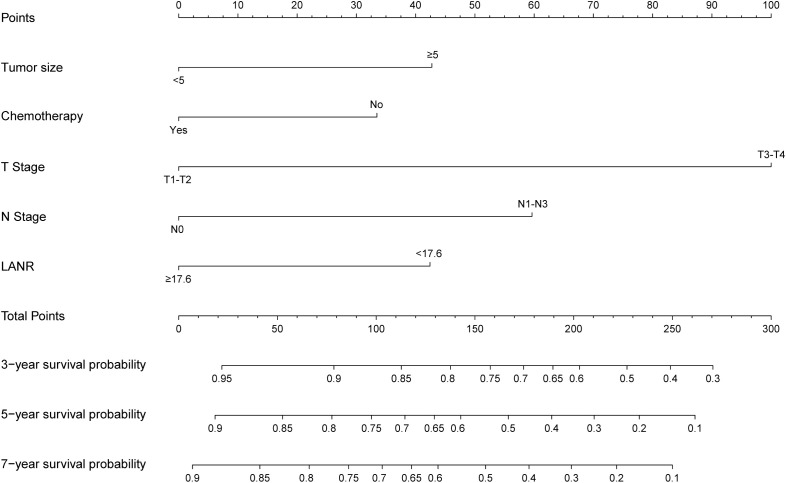
The nomogram for 3-, 5-, and 7-year OS in patients with GC.

**Figure 3:**
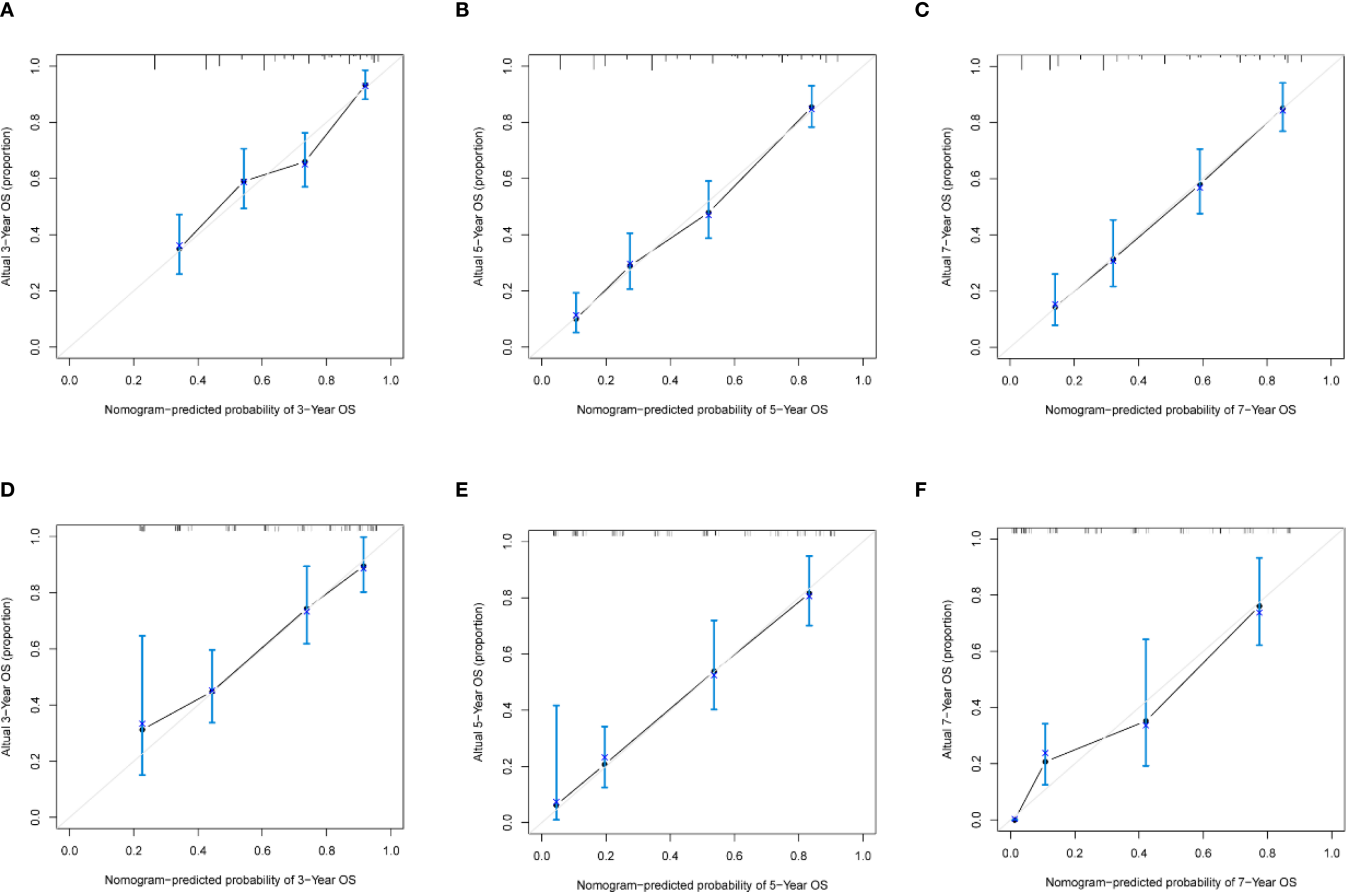
The calibration curves of the nomogram. Calibration curves of 3-, 5-, 7-year OS in the training set **(A–C)**; and the validation set **(D–F)**.

**Figure 4:**
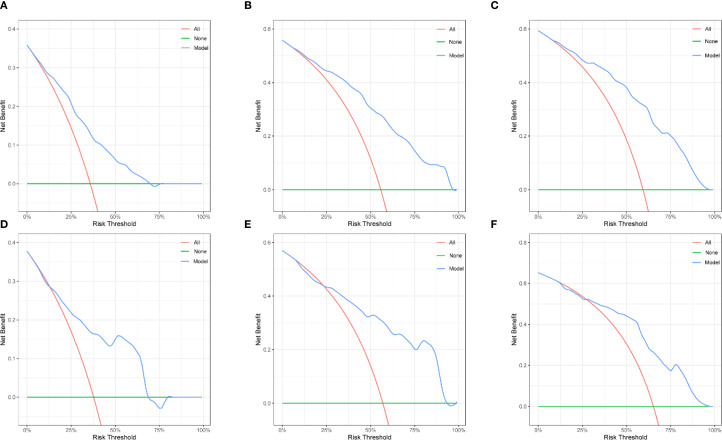
The decision curve analyses of the nomogram. Decision curve analyses of 3-, 5-, 7-year OS in the training set **(A–C)**; and the validation set **(D–F)**.

**Figure 5:**
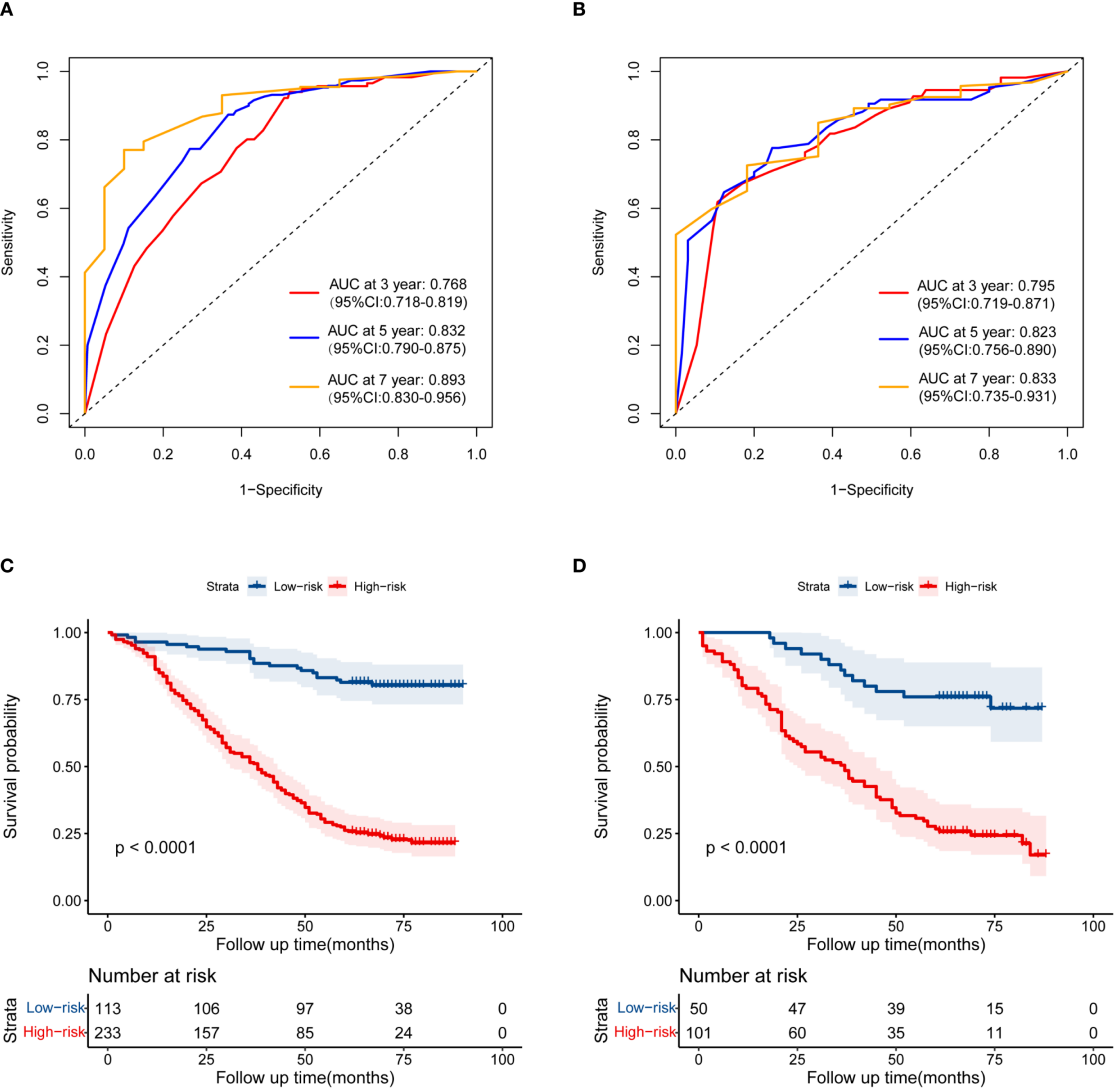
The receiver operating characteristic curves of the nomogram predicting 3-, 5- and 7-year OS in the training cohort **(A)** and the validation cohort **(B)**. Kaplan-Meier curves of OS for the low-risk and high-risk groups in the training cohort **(C)** and the validation cohort **(D)**.

Based on the independent prognostic factors identified by multivariate Cox regression analysis, a nomogram model for the training cohort was constructed (**Figure 2**). The C-index of the nomogram was 0.726 (95% Confidence Interval [CI] =0.688–0.758). The calibration curve demonstrated excellent agreement between the predicted and observed outcomes (**Figures 3A–C**). Additionally, the DCA validated the clinical utility of the nomogram (**Figures 4A–C**). The AUC values for predicting 3-year, 5-year, and 7-year OS were 0.768(95% CI = 0.718–0.819), 0.832(95% CI = 0.790–0.875) and 0.893(95% CI = 0.830–0.956), respectively (**Figure 5A**).

In the validation cohort, the C-index was 0.745(95% CI = 0.687–0.787), Calibration curves and DCA are shown in **Figures 3D–F** and **Figures 4D–F**. The AUC values for predicting 3-year, 5-year, and 7-year OS in the validation cohort were 0.795(95% CI = 0.719–0.871), 0.823(95% CI = 0.756–0.890) and 0.833(95% CI = 0.735–0.931), respectively (**Figure 5B**).

### Risk stratification of the nomogram

The total score was calculated based on the nomogram model, and the optimal cutoff value was determined to be 127.0 using X-tile software. Patients were classified into high-risk and low-risk groups based on this cutoff value. Kaplan-Meier curves showed that in both the training cohort (**Figure 5C**) and the validation cohort (**Figure 5D**), patients in the low-risk subgroup had significantly better survival outcomes compared to those in the high-risk subgroup.

## Discussion

The incidence and mortality of GC are continually rising, significantly impacting human life and health. Despite the use of various factors, such as TNM staging, for prognostic evaluation, the risks of recurrence and death remain unclear. Inflammation, immune status, and nutritional condition have increasingly drawn attention in the pathogenesis and prognosis of GC. Integrating these three factors to comprehensively assess the prognosis of GC and provide treatment guidance is of great significance.

Inflammation is closely linked to tumors and plays a crucial role in tumorigenesis, progression, and metastasis (18). Neutrophils, important leukocytes in the immune system, exert a dual role within the tumor microenvironment. They promote tumor growth, angiogenesis, and metastasis by secreting cytokines, chemokines, and proteases (19). Simultaneously, they directly kill tumor cells by releasing reactive oxygen species and nitrogen oxides, or indirectly enhance the immune system’s attack on tumors by promoting T cell activity (20). Neutrophils can also secrete inhibitory cytokines that suppress T cell and natural killer cell functions, leading to immune escape and tumor progression (21, 22). Additionally, neutrophils can promote tumor metastasis by forming neutrophil extracellular traps that capture and assist tumor cells in colonizing new environments (23). Lymphocytes, major components of the tumor immune barrier, directly contribute to the killing of tumor cells and inhibit tumor cell proliferation and migration by secreting cytokines, inducing cytotoxic cell death, and playing a key role in tumor immune surveillance (24). Hematological biomarkers associated with neutrophils and lymphocytes have been shown to be independent prognostic factors in cancer patients, effectively predicting patient prognosis (25–27). Serum albumin, one of the most abundant proteins in the blood, plays a key role in maintaining plasma colloid osmotic pressure and overall nutrition and is commonly used in clinical assessments of nutritional status (28). In addition, serum albumin has been implicated tumor immune escape, growth, and metastasis. Nutrition is closely linked to the immune system, and malnutrition can activate systemic inflammation, impairing immune function and affecting the prognosis of cancer patients (9, 29). Studies have shown that serum albumin is an independent risk factor for the prognosis of various malignancies, including cardia adenocarcinoma (30), nasopharyngeal carcinoma (31), kidney cancer (32), and head and neck cancer (33). Therefore, changes in serum albumin levels provide important prognostic information for clinical decision-making, aiding in the evaluation of treatment outcomes and prognosis in cancer patients.

As an integrative metric, LANR captures three key biological dimensions of host–tumor interaction. Lower LANR values—reflecting neutrophilia, lymphopenia, and hypoalbuminemia—may mark a high−risk host–tumor milieu characterized by systemic inflammation, impaired cellular immunity, and reduced tolerance to therapy. Such a milieu may facilitate tumor invasion, metastasis, and immune evasion. These mechanisms could partly explain the poorer survival observed in our cohort. Conversely, higher LANR values indicate preserved immune surveillance, attenuated systemic inflammation, and adequate nutritional reserves, consistent with better outcomes. Our study found that LANR is a strong prognostic indicator for GC patients, consistent with previous research findings.

In this study, we developed a novel index, LANR, which comprehensively reflects inflammation, immunity, and nutrition. This index is more comprehensive than single markers of immunity, inflammation, or nutrition alone. To our knowledge, this is the first study to investigate the prognostic value of LANR in GC patients. Several clinical studies have confirmed the prognostic significance of LANR. For instance, Wang et al. (34) found that LANR can predict relapse-free survival in endometrial cancer. Zhuang et al. (14) demonstrated that LANR is a reliable predictor of OS in resectable pancreatic ductal adenocarcinoma. Our study shown that lower preoperative LANR levels are associated with poor prognosis in GC patients, and that LANR is an independent prognostic factor for OS in GC patients. The associations between LANR and clinicopathological features differed between the two cohorts, possibly reflecting differences in sample size and baseline characteristics. In addition, the independent prognostic performance of LANR is superior compared to NLR, PNI and PLR. By integrating LANR with clinical and pathological parameters, we constructed a nomogram to predict 3-year, 5-year, and 7-year OS in GC patients, visually illustrating the impact of immunity, inflammation, and nutrition on survival outcomes in these patients. Calibration curves demonstrated good agreement between predicted and observed survival and DCA indicated net benefit across a range of clinically plausible threshold probabilities, supporting the nomogram’s reliability and potential clinical utility. The model may mitigate limitations of TNM staging by providing individualized risk estimates that complement stage-based categories. As an adjunctive risk-stratification tool, the nomogram may identify high-risk patients and guide closer surveillance and tailored management.

However, this study has several limitations. First, it is a single-center retrospective study with a limited sample size, which may introduce bias into the results. Therefore, large-scale, multicenter prospective studies are needed in the future to further validate these findings. Second, although we established a validation cohort for internal validation, external validation is still lacking. In subsequent studies, we plan to conduct multicenter external validation to enhance the reliability and generalizability of our findings.

## Conclusion

In conclusion, our study demonstrates that preoperative LANR is an independent prognostic factor for GC patients. Based on LANR, we developed a new nomogram model that demonstrates high accuracy and potential clinical utility. Clinicians can use risk stratification to identify high-risk patients and tailor personalized interventions, providing scientific guidance and recommendations for the treatment of gastric cancer patients.

## Data Availability

The original contributions presented in the study are included in the article/**Supplementary Material**. Further inquiries can be directed to the corresponding author.

## References

[1] Bray F Laversanne M Sung H Ferlay J Siegel RL Soerjomataram I. Global cancer statistics 2022: GLOBOCAN estimates of incidence and mortality worldwide for 36 cancers in 185 countries. CA Cancer J Clin. (2024) 74:229–63. doi: 10.3322/caac.21834, PMID: 38572751

[2] Lordick F Carneiro F Cascinu S Fleitas T Haustermans K Piessen G. Gastric cancer: ESMO Clinical Practice Guideline for diagnosis, treatment and follow-up. Ann Oncol. (2022) 33:1005–20. doi: 10.1016/j.annonc.2022.07.004. Electronic address: clinicalguidelines@esmo.org., PMID: 35914639

[3] Wang F-H Zhang X-T Li Y-F Tang L Qu X-J Ying J-E. The Chinese Society of Clinical Oncology (CSCO): Clinical guidelines for the diagnosis and treatment of gastric cancer, 2021. Cancer Commun (Lond). (2021) 41:747–95. doi: 10.1002/cac2.12193, PMID: 34197702 PMC8360643

[4] Mullaney PJ Wadley MS Hyde C Wyatt J Lawrence G Hallissey MT. Appraisal of compliance with the UICC/AJCC staging system in the staging of gastric cancer. Union Internacional Contra la Cancrum/American Joint Committee on Cancer. Br J Surg. (2002) 89:1405–8. doi: 10.1046/j.1365-2168.2002.02262.x, PMID: 12390382

[5] Gao X Pan Y Han W Hu C Wang C Chen L. Association of systemic inflammation and body mass index with survival in patients with resectable gastric or gastroesophageal junction adenocarcinomas. Cancer Biol Med. (2021) 18:283–97. doi: 10.20892/j.issn.2095-3941.2020.0246, PMID: 33628601 PMC7877168

[6] Murata M. Inflammation and cancer. Environ Health Prev Med. (2018) 23:50. doi: 10.1186/s12199-018-0740-1, PMID: 30340457 PMC6195709

[7] Tan CSY Read JA Phan VH Beale PJ Peat JK Clarke SJ. The relationship between nutritional status, inflammatory markers and survival in patients with advanced cancer: a prospective cohort study. Support Care Cancer. (2015) 23:385–91. doi: 10.1007/s00520-014-2385-y, PMID: 25112562

[8] Hanahan D Weinberg RA. Hallmarks of cancer: the next generation. Cell. (2011) 144:646–74. doi: 10.1016/j.cell.2011.02.013, PMID: 21376230

[9] McGovern J Dolan RD Skipworth RJ Laird BJ McMillan DC. Cancer cachexia: a nutritional or a systemic inflammatory syndrome? Br J Cancer. (2022) 127:379–82. doi: 10.1038/s41416-022-01826-2, PMID: 35523879 PMC9073809

[10] Tan S Zheng Q Zhang W Zhou M Xia C Feng W. Prognostic value of inflammatory markers NLR, PLR, and LMR in gastric cancer patients treated with immune checkpoint inhibitors: a meta-analysis and systematic review. Front Immunol. (2024) 15:1408700. doi: 10.3389/fimmu.2024.1408700, PMID: 39050856 PMC11266030

[11] Wu H Zhang J Zhou B Ma S Zheng Y. Preoperative monocyte to high-density lipoprotein ratio as a predictor of survival outcome of gastric cancer patients after radical resection. biomark Med. (2023) 17:123–31. doi: 10.2217/bmm-2022-0691, PMID: 37042447

[12] Aoyama T Maezawa Y Hashimoto I Hara K Tamagawa A Kazama K. The CRP-albumin-lymphocyte (CALLY) index is an independent prognostic factor for gastric cancer patients who receive curative treatment. Anticancer Res. (2024) 44:1629–36. doi: 10.21873/anticanres.16961, PMID: 38537973

[13] Christodoulidis G Voutyras A Fotakopoulos G Koumarelas K-E Georgakopoulou VE Kouliou M-N. CRP to albumin ratio as a prognostic nutrition-based biomarker for patients with gastric cancer: A narrative review. Cureus. (2024) 16:e71516. doi: 10.7759/cureus.71516, PMID: 39553069 PMC11563776

[14] Zhuang J Wang S Wang Y Wu Y Hu R. Prognostic significance of preoperative lymphocytes, albumin, and neutrophils (LANR) index in resectable pancreatic ductal adenocarcinoma. BMC Cancer. (2024) 24:568. doi: 10.1186/s12885-024-12329-z, PMID: 38714979 PMC11075219

[15] Zhang S Chen Z Ling J Feng Y Xie Y Liu X. Nomograms based on the lymphocyte-albumin-neutrophil ratio (LANR) for predicting the prognosis of nasopharyngeal carcinoma patients after definitive radiotherapy. Sci Rep. (2024) 14:5388. doi: 10.1038/s41598-024-56043-z, PMID: 38443675 PMC10915143

[16] Liang X Yao S Lu P Ma Y Xu H Yin Z. The prognostic value of new index (LANR) composed of pre-operative lymphocytes, albumin, and neutrophils in patients with resectable colorectal cancer. Front Oncol. (2021) 11:610264. doi: 10.3389/fonc.2021.610264, PMID: 34150609 PMC8210780

[17] Camp RL Dolled-Filhart M Rimm DL. X-tile: a new bio-informatics tool for biomarker assessment and outcome-based cut-point optimization. Clin Cancer Res. (2004) 10:7252–9. doi: 10.1158/1078-0432.CCR-04-0713, PMID: 15534099

[18] Greten FR Grivennikov SI. Inflammation and cancer: triggers, mechanisms, and consequences. Immunity. (2019) 51:27–41. doi: 10.1016/j.immuni.2019.06.025, PMID: 31315034 PMC6831096

[19] Xiong S Dong L Cheng L. Neutrophils in cancer carcinogenesis and metastasis. J Hematol Oncol. (2021) 14:173. doi: 10.1186/s13045-021-01187-y, PMID: 34674757 PMC8529570

[20] Quail DF Amulic B Aziz M Barnes BJ Eruslanov E Fridlender ZG. Neutrophil phenotypes and functions in cancer: A consensus statement. J Exp Med. (2022) 219:e20220011. doi: 10.1084/jem.20220011, PMID: 35522219 PMC9086501

[21] Tamura K Miyato H Kanamaru R Sadatomo A Takahashi K Ohzawa H. Activated neutrophils inhibit chemotactic migration of activated T lymphocytes to CXCL11 by multiple mechanisms. Cell Immunol. (2023) 384:104663. doi: 10.1016/j.cellimm.2023.104663, PMID: 36638767

[22] Petersson J Askman S Pettersson Å Wichert S Hellmark T Johansson ÅCM. Bone marrow neutrophils of multiple myeloma patients exhibit myeloid-derived suppressor cell activity. J Immunol Res. (2021) 2021:6344344. doi: 10.1155/2021/6344344, PMID: 34414242 PMC8369183

[23] Ma Y Wei J He W Ren J. Neutrophil extracellular traps in cancer. MedComm (2020). (2024) 5:e647. doi: 10.1002/mco2.647, PMID: 39015554 PMC11247337

[24] Kyrysyuk O Wucherpfennig KW. Designing cancer immunotherapies that engage T cells and NK cells. Annu Rev Immunol. (2023) 41:17–38. doi: 10.1146/annurev-immunol-101921-044122, PMID: 36446137 PMC10159905

[25] Faria SS Giannarelli D Cordeiro de Lima VC Anwar SL Casadei C De Giorgi U. Development of a prognostic model for early breast cancer integrating neutrophil to lymphocyte ratio and clinical-pathological characteristics. Oncologist. (2024) 29:e447–54. doi: 10.1093/oncolo/oyad303, PMID: 37971409 PMC10994264

[26] Shen X Xiang M Tang J Xiong G Zhang K Xia T. Evaluation of peripheral blood inflammation indexes as prognostic markers for colorectal cancer metastasis. Sci Rep. (2024) 14:20489. doi: 10.1038/s41598-024-68150-y, PMID: 39227608 PMC11372090

[27] Liu J Sun R Cai K Xu Y Yuan W. A nomogram combining neutrophil to lymphocyte ratio (NLR) and prognostic nutritional index (PNI) to predict distant metastasis in gastric cancer. Sci Rep. (2024) 14:15391. doi: 10.1038/s41598-024-65307-7, PMID: 38965325 PMC11224267

[28] Tomasiewicz A Polański J Tański W. Advancing the understanding of malnutrition in the elderly population: current insights and future directions. Nutrients. (2024) 16:2502. doi: 10.3390/nu16152502, PMID: 39125381 PMC11314143

[29] Mecca M Picerno S Cortellino S. The killer’s web: interconnection between inflammation, epigenetics and nutrition in cancer. Int J Mol Sci. (2024) 25:2750. doi: 10.3390/ijms25052750, PMID: 38473997 PMC10931665

[30] Lien Y-C Hsieh C-C Wu Y-C Hsu H-S Hsu W-H Wang L-S. Preoperative serum albumin level is a prognostic indicator for adenocarcinoma of the gastric cardia. J Gastrointest Surg. (2004) 8:1041–8. doi: 10.1016/j.gassur.2004.09.033, PMID: 15585392

[31] Yang H Wang K Liang Z Guo S Zhang P Xu Y. Prognostic role of pre-treatment serum albumin in patients with nasopharyngeal carcinoma: A meta-analysis and systematic review. Clin Otolaryngol. (2020) 45:167–76. doi: 10.1111/coa.13454, PMID: 31573757

[32] Chen Z Shao Y Wang K Cao W Xiong Y Wu R. Prognostic role of pretreatment serum albumin in renal cell carcinoma: a systematic review and meta-analysis. Onco Targets Ther. (2016) 9:6701–10. doi: 10.2147/OTT.S108469, PMID: 27822073 PMC5094571

[33] Danan D Shonka DC Selman Y Chow Z Smolkin ME Jameson MJ. Prognostic value of albumin in patients with head and neck cancer. Laryngoscope. (2016) 126:1567–71. doi: 10.1002/lary.25877, PMID: 26864349

[34] Wang Y Zheng Y Tian C Yu J Rao K Zeng N. Nomogram based on immune-inflammatory score and classical clinicopathological parameters for predicting the recurrence of endometrial carcinoma: A large, multi-center retrospective study. J Inflammation Res. (2024) 17:11437–49. doi: 10.2147/JIR.S494716, PMID: 39735898 PMC11675361

