# Supplementary material for: Nomogram based on the novel index LANR, composed of preoperative lymphocytes, albumin, and neutrophils, for predicting prognosis in patients with gastric cancer: a retrospective study
Source: Front Oncol. 2025 Sep 29;15:1634948. doi: 10.3389/fonc.2025.1634948 (PMC12515683; doi:10.3389/fonc.2025.1634948)
Supplement: Supplementary file 1 [file Table1.docx]

**Supplementary TABLE 1** Comparison of the independent predictive performance of LANR and other indices in the training cohort

| Variables | 3 years | | 5 years | | 7 years | |
| --- | --- | --- | --- | --- | --- | --- |
|  | AUC | 95%CI | AUC | 95%CI | AUC | 95%CI |
| LANR | 0.569 | (0.505,0.633) | 0.645 | (0.587,0.704) | 0.719 | (0.613,0.826) |
| NLR | 0.546 | (0.482,0.610) | 0.623 | (0.564,0.682) | 0.692 | (0.577,0.808) |
| PNI | 0.604 | (0.541,0.667) | 0.641 | (0.583,0.700) | 0.654 | (0.534,0.774) |
| PLR | 0.524 | (0.459,0.590) | 0.585 | (0.525,0.646) | 0.711 | (0.601,0.821) |
