# Supplementary material for: Nomogram based on the novel index LANR, composed of preoperative lymphocytes, albumin, and neutrophils, for predicting prognosis in patients with gastric cancer: a retrospective study
Source: Front Oncol. 2025 Sep 29;15:1634948. doi: 10.3389/fonc.2025.1634948 (PMC12515683; doi:10.3389/fonc.2025.1634948)
Supplement: Supplementary file 2 [file Table2.docx]

**Supplementary TABLE 2** Comparison of the independent predictive performance of LANR and other indices in the validation cohort

| Variables | 3 years | | 5 years | | 7 years | |
| --- | --- | --- | --- | --- | --- | --- |
|  | AUC | 95%CI | AUC | 95%CI | AUC | 95%CI |
| LANR | 0.649 | (0.560,0.739) | 0.609 | (0.517,0.700) | 0.611 | (0.427,0.796) |
| NLR | 0.630 | (0.540,0.721) | 0.589 | (0.497,0.681) | 0.590 | (0.402,0.778) |
| PNI | 0.588 | (0.493,0.684) | 0.599 | (0.508,0.691) | 0.556 | (0.396,0.717) |
| PLR | 0.626 | (0.529,0.723) | 0.619 | (0.529,0.709) | 0.578 | (0.398,0.758) |
